# Biology and external morphology of the immature stages of the butterfly *Callicore pygas eucale* , with comments on the taxonomy of the genus *Callicore* (Nymphalidae: Biblidinae)

**DOI:** 10.1093/jis/14.1.91

**Published:** 2014-07-17

**Authors:** Fernando Maia Silva Dias, Mirna Martins Casagrande, Olaf Hermann Hendrik Mielke

**Affiliations:** 1 Laboratório de Estudos de Lepidoptera Neotropical, Departamento de Zoologia, Universidade Federal do Paraná, P.O. Box 19.020, ZIP Code 81.531-980, Curitiba, Paraná, Brazil

**Keywords:** Callicorina, *Allophylus*, Sapindaceae

## Abstract

The biology and the external morphology of the immature stages of *Callicore pygas eucale* (Fruhstorfer, 1916) (Lepidoptera: Nymphalidae: Biblidinae) are described. Immatures were collected on *Allophylus edulis* (Radlkofer) (Sapindales: Sapindaceae) in Curitiba, Paraná, Brazil, and reared in the laboratory. Morphological descriptions and illustrations are given based on observations through electronic, stereoscopic, and optic microscopes, the latter two attached to a camera lucida. Results are compared and discussed with the immature stages of other species of the subtribe Callicorina. Immature stages data provide further evidence that *Callicore* is paraphyletic and that generic limits within the Callicorina need revision.

## Introduction

**Fig 1-4.**
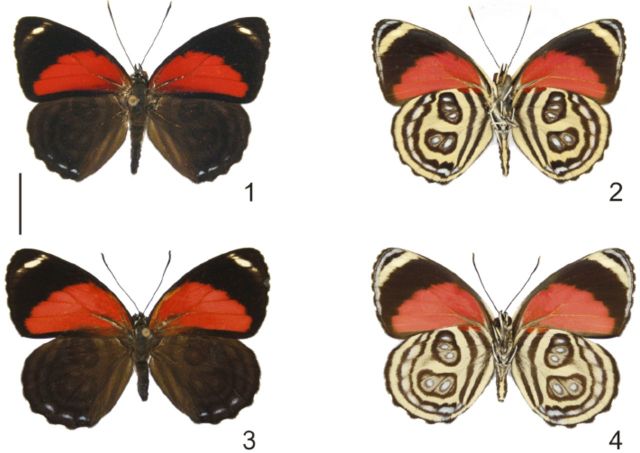
*Callicore pygas eucale* (Fruhstorfer, 1916). 1. Male, dorsal. 2. Male, ventral. 3. Female, dorsal. 4. Female, ventral. Scale bar = 1cm.

The Neotropical genus *Callicore* Hübner [1819] is a group of small to medium sized butterflies, many of which are recognized by the numerical patterns on the hind wing underside. *Callicore* is the largest genus in the subtribe Callicorina (Callicorini *sensu* Freitas and Brown 2004), with 91 recognized taxa in 20 species ( Lamas 2004 ). Species of *Callicore* are widely distributed throughout Central and South America, with the greatest diversity in the eastern slope of the Andes and the Amazon basin ( DeVries 1987 ). *Callicore pygas* (Godart [1824]) is a widespread species, ranging from the western slope of the Andes, through the Amazon Basin and the Atlantic Forest, with eight recognized subspecies. Males and females are similar: black ground color on the upper side of both wings, forewing with a red basal patch and small white apical spots, hind wing with small bluish marginal spots; underside of the forewing similar to the upper side, but with additional yellow and bluish apical and marginal markings; hind wing underside with two groups of two and three blue dots, surrounded by alternating yellow and black lines in concentric circles. The taxon with the southernmost distribution among all *C pygas* subspecies is *C pygas eucale* (Fruhstorfer, 1916) (Lepidoptera: Nymphalidae: Biblidinae) ( Figures 1-4 ), inhabiting the Atlantic and Araucaria forests in the Brazilian states of Paraná, Santa Catarina, and Rio Grande do Sul ( D’Abrera 1987 ). As noted by Descimon (1986) , DeVries (1987) , and Neild (1996) for other species of the genus, *C pygas eucale* is strikingly similar to a small-sized *Agrias* Doubleday, 1845 (Nymphalidae: Charaxinae). *Agrias claudina annetta* (Gray, 1832) is the only taxon of the genus sympatric with *C pygas eucale* ( Lamas 2004 ).

Immature stages of *C pygas eucale* were briefly described by Müller (1886) , who reported great similarity with *Diaethria clymena* (Cramer, [1775]), a species recently described in detail by Barbosa et al. (2010) . The immature stages of an additional 10 taxa of *Callicore* were briefly described and/or illustrated by Freitas (1999) , DeVries (1987) , Teshirogi (2007) , and Janzen and Hallwachs (2012). The host plants are all in the Sapindaceae family, including a number of species of *Allophylus, Serjania, Paullinia,* and *Urvillea* (Beccaloni et al. 2009).

As discussed by Barbosa et al. (2010) and Dias et al. (2012) , it is important to note that the taxonomic limits of many members of the Callicorina are still unclear and that immature morphology and host plant use will likely provide phylogenetically important information, not only within the subtribe but also within higher taxonomic groups. This paper describes the biology and the external morphology of *C pygas eucale* and discusses its biology and morphology with other species of *Callicore* and species of related genera in the tribe Callicorina.

## Materials and Methods

Specimens were collected on several occasions between May 2008 and May 2009 at Parque Municipal Barigui, municipality of Curitiba, State of Paraná, Brazil (25°25’36”S, 49°18’32”W) c.a. 950 m a.s.l. Collected specimens were brought to the Laboratório de Estudos de Lepidoptera Neotropical, Departamento de Zoologia, Universidade Federal do Paraná, and reared at ambient conditions. Therefore, the durations of life-stages reported here may not correspond exactly with natural lifecycle durations, as laboratory conditions do not necessarily match those of the areas where larvae were collected. Rearing methodology was as described by Dias et al. (2012) , with minor modifications: specimens were reared individually in plastic containers, and old leaves of the host plant were changed for fresher ones as necessary. The plastic containers were examined daily to observe instars’ changes and behavior, and also to control the moisture inside. Behavioral observations were carried out in the field as well as in the laboratory. Eggs and head capsules were dehydrated and preserved; larvae and pupae were fixed in Kahle-Dietrich solution and preserved in 80% alcohol. Eggs were analyzed in an electron scanning microscope; the chaetotaxy of the head capsule was observed using an optic microscope equipped with a camera lucida. Measurements and drawings of head capsules were made with the aid of a stereoscopic microscope equipped with micrometric lenses or a camera lucida; width of the head capsule is given at the height of the second stemma in anterior view. Nomenclature follows Scoble (1992) for eggs; Hinton (1946) , Peterson (1962) , and Stehr (1987) for larval chaetotaxy and morphology, with modifications proposed by Huertas-Dionisio (2006) for the chaetotaxy of the anal prolegs; and Mosher (1916) for pupal morphology. Voucher specimens are retained at the Coleção de Imaturos de Lepidoptera (DZUPIL), Coleção Entomológica Pe. Jesus Santiago Moure, Departamento de Zoologia, Universidade Federal do Paraná.

## Results

### General observations

**Fig 5-20.**
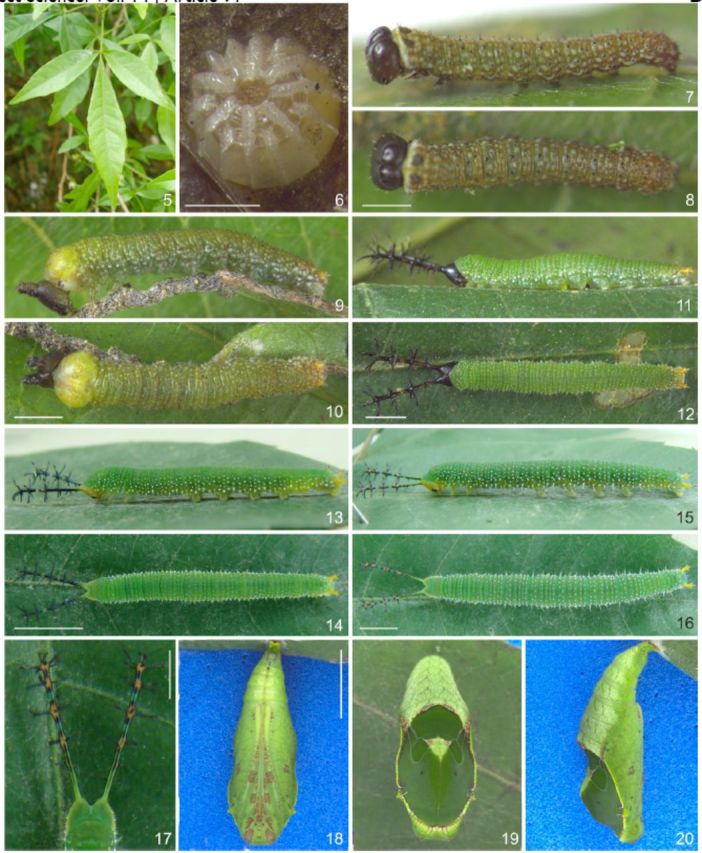
Immature stages and host plant of *Callicore pygas eucale* (Fruhstorfer, 1916). 5. Host plant, *Allophylus edulis.* 6. Egg, dorsal. 7–8. First instar. 7. Lateral. 8. Dorsal. 9–10. Second instar. 9. Lateral. 10. Dorsal. 11–12. Third instar. 11. Lateral. 12. Dorsal. 13–14. Fourth instar. 13. Lateral. 14. Dorsal. 15–16. Fifth instar. 15. Lateral. 16. Dorsal. 17. Fifth instar, detail of the head capsule scoli, dorsal. 18–20. Pupa. 18. Lateral. 19. Dorsal. 20. Ventral. Scale bars: Figures 6–8 = 0.5mm; Figures 9–10 = 1mm; Figures 11–12 = 2mm; Figures 13–16 = 0.5cm; Figure 17 = 0.25cm; Figures 18–20 = 0.5cm.

Females of *C. pygas eucale* lay eggs on *Allophylus edulis* (Radlkofer) (Sapindales: Sapindaceae) ( Figure 5 ), a tree commonly found at the study site along forest and trail edges and in forest gaps. Eggs were found one time on *Allophylus puberulus,* and host plant shift experiments carried out in the laboratory also confirm the use of a number of species of *Serjania* by *C. pygas eucale. Allophylus petio-lulatus,* cited by Müller (1886) and Beccaloni et al. (2009) for *C. pygas eucale,* was not found at the study site. Eggs are laid singly on the underside of mature leaves, close to the tip. The darkening head of the developing larva becomes visible through the chorion a few days prior to eclosion. First and second instars build frass chains in one of the three folioles (i.e., leaflets) of the host plant with silk and fecal pellets, extending the primary vein considerably beyond the leaf margin, resting with the head capsule towards the leaf margin. Third to fifth instars rest with the head frons and scoli parallel to the substrate, against the leaf surface and with the head directed towards the petiole. Later instars wobble the head capsule or the anterior part of the body vigorously when disturbed, secreting a translucent green liquid through the adenosma or jugular gland ( Vegilante and Hasenfuss 2012 ). Before the change of instars, the head capsule is always visible through the larval skin as a yellow growth between the head and the thorax ( Figures 9-10 ). Before pupation, larvae stop eating, become dull-colored and considerably swollen, and begin a wandering phase. Upon finding a place to pupate, larvae spin a silk pad on the upper surface of a host plant leaf and attach their anal prolegs to this pad, remaining stretched face down on the upper surface of the leaf until molt. Pupae are capable of vigorous movement and thrash violently if disturbed by flexing the posterior abdominal segments side to side.

### Morphology

**Fig 21-23.**
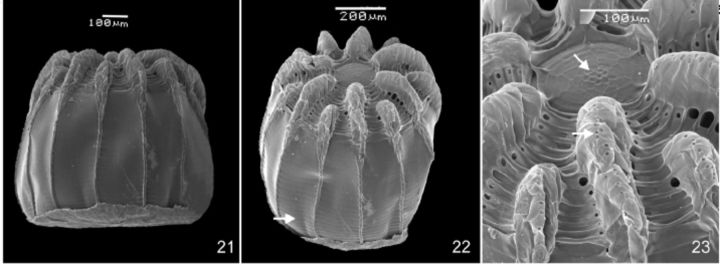
Egg of *Callicore pygas eucale* (Fruhstorfer, 1916). 21. Lateral. 22. Dorsal, arrow indicates horizontal ridges. 23. Detail of the aeropyla (lower arrow) on the vertical ridge and the sculptured area around micropyla (upper arrow), dorsal.

Egg ( Figures 6 , 21-23): yellow; barrel shaped, with flattened base and apex; with 14 vertical and 40-44 horizontal ridges. Vertical ridges symmetrically arranged around a circular flattened area, alternating larger and smaller projections close to the apex, forming a ‘crown’ ( Figure 22 ); flattened area with rosette-like sculptures ( Figure 23 ). Horizontal ridges noticeable from the base to the apex of the egg, with 12-14 more distinct ridges near the apical area ( Figure 22 ); aeropylae on the apical projections of the vertical ridges ( Figure 23 ). Approximate duration: 3-5 days (average 4 days, n = 4). Average diameter 0.76 mm (SD = 0.002); and height 0.6 mm (SD = 0.003) (n = 2).

**Fig 24-28.**
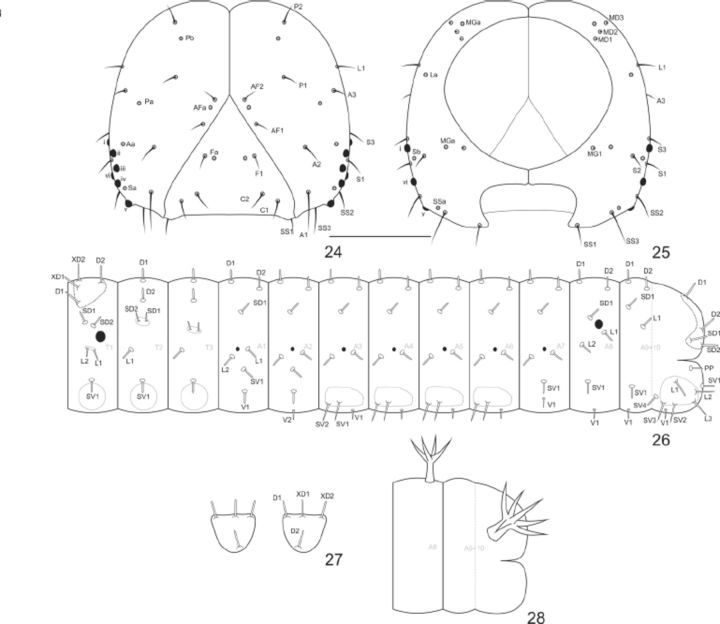
Chaeototaxy and larval structures of *Callicore pygas eucale* (Fruhstorfer, 1916). 24–25. First instar head capsule. 24. Anterior. 25. Posterior. 26. Thorax and abdomen schematic representation, lateral. 27. Prothoracic plate, dorsal. 28. A8 and A9+10, schematic representation of the scoli on the fifth instar. Scale bar: Figures 24–25 = 0.25mm.

First instar ( Figures 7-8 , 24-27 ): head capsule dark brown, smooth and slightly bilobated along the epicranial suture; six stemmata roughly placed in a semicircle ( Figures 24-25 ); labrum bilobated and mandibles with round teeth. Body mostly dark green; yellowish-green on the prothorax and A8-A9+10; with whitish spots scattered ventral to the spiracular area; and dark brown setae inserted on roundish dark brown pinnacula dorsal to the spiracular area. Prothoracic dorsal plate dark brown and semicircular ( Figure 27 ); adenosma ventral, between the head capsule and the prothorax; suranal plate “Y” shaped, weakly sclerotized; thoracic and abdominal leg plates and ocrea dark brown, abdominal and anal legs bearing 10-12 uniordinal and uniserial crochets arranged in a lateral penellipse. Chaetotaxy of the head capsule and body are given by figures 24-27 . Approximate duration: 3-9 days (average 4.5 days, n = 4). Average head capsule width: 0.59 mm (SD = 0.043) (n = 4).

**Fig 29-30.**
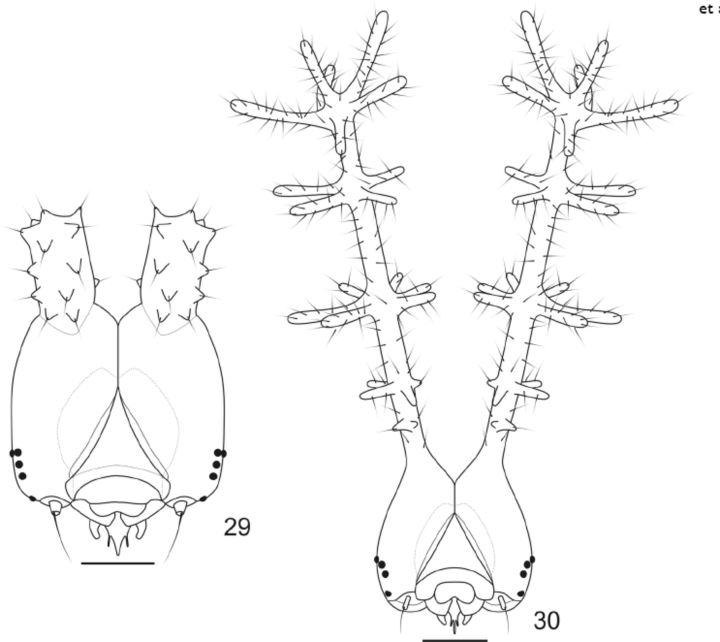
Head capsules of *Callicore pygas eucale* (Fruhstorfer, 1916), anterior. 29. Second instar. 30. Third instar. Scale bars: Figure 29 = 0.25mm; Figures 30 = 0.5mm.

Second instar ( Figures 9-10 , 29 ): head capsule dark brown, frons and adjacent area yellowish, with a pair of thick truncated scoli with numerous small spines, about half the height of the head capsule, one on each side of the epicranial suture ( Figure 29 ). Body dorsal, subdorsal and supraspiracular areas dark green; subspiracular subventral and ventral areas green; with scattered whitish spots on the insertion of the secondary setae; prothoracic plate green; thoracic and abdominal leg plates and ocrea dark green; A9+10 yellowish-green, with a pair of small and bright yellow scoli; suranal plate indistinct. Approximate duration: 3-8 days (average 5 days, n = 5). Average head capsule width: 0.72 mm (SD = 0.043) (n = 6).

Third instar ( Figures 11-12 , 30 ): head capsule mostly dark brown, frons yellowish; with scattered tiny projections and with slender and branched scoli about three times the height of the head capsule, one on each side of the epicranial suture. Scoli covered by long bristles and tiny anterior spines near the base of the shaft; four rows of radial spines on the first (four smaller spines), second (six spines) and third (four spines) fourth of the shaft of the scoli, which ends in a “crown” ( Barbosa *et al.* 2010 ) of five spines and an additional distal spine ( Figure 30 ). Shaft of the scoli yellowish between the second and third rows of spines. Body entirely green but lighter green ventrally, with scattered whitish spots on the insertion of the secondary setae and along the spiracular area; prothoracic plate of the same color as the surrounding areas; thoracic and abdominal leg plates and ocrea translucent light green; A8 with a dorsal two to four branched scolus, green and with the tip of the spines black; A9+10 with a pair of five branched scoli, yellow and with the tip of the spines black. Approximate duration: 6-8 days (average 6.8 days, n = 9). Average head capsule width: 1.19 mm (SD = 0.042) (n = 10).

**Fig 31-32.**
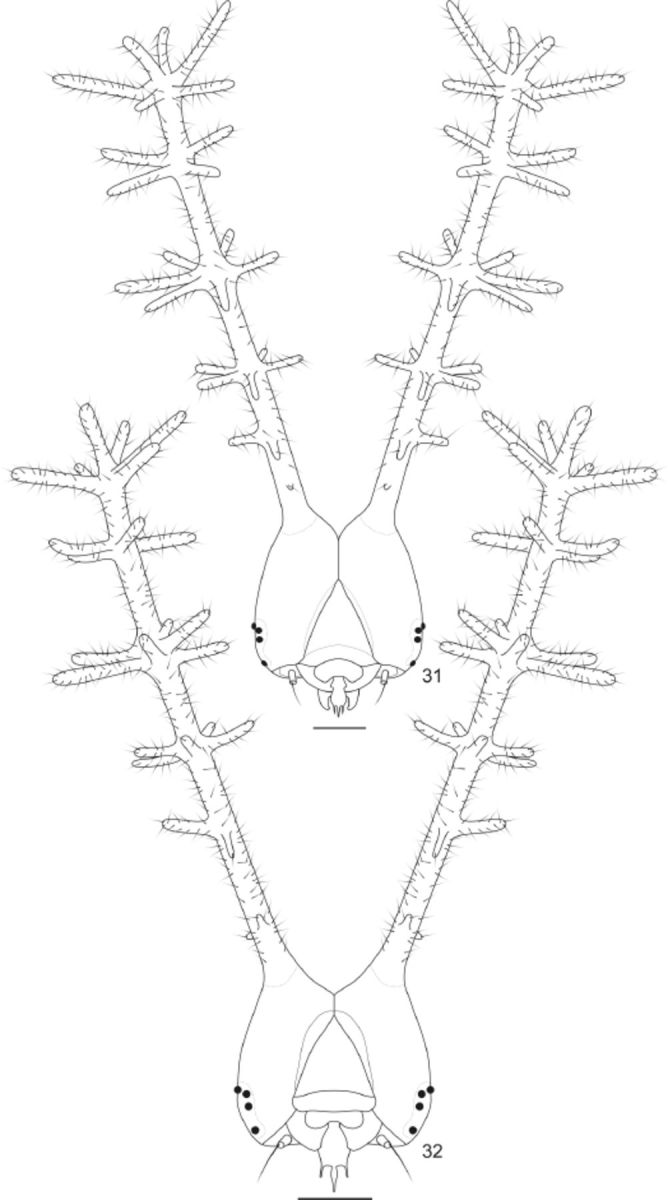
Head capsule of *Callicore pygas eucale* (Fruhstorfer, 1916), anterior. 31. Fourth instar. 32. Fifth instar. Scale bar: Figure 31 = 0.5mm; fig 32 = 0.1cm

Fourth instar ( Figures 13-14 , 31 ): head capsule bright yellow, except for the area around the stemata, dark brown, and the dorso-posterior area, green; scoli about three times and a half the height of the head capsule; base of the scoli with a tiny anterior spines, a single row of anterior spines on the first fifth of the shaft of the scoli (two anterior smaller spines), and four rows of radial spines in the second (five spines), third (six spines) and fourth (four spines) fifths of the shaft of the scoli; “crown” similar to the previous instar ( Figure 31 ); shaft of the scoli yellowish between the first and the second, second and third and third and fourth rows of spines, so as the mid portion of the spines. Body generally similar in color and shape to the previous instar, but brighter green in color, and spots on the insertion of the secondary setae and along the spiracular area silvery-white; scoli on A8 and A9+10 strongly developed. Approximate duration: 4-22 days (average 8.5 days, n = 15). Average head capsule width: 1.57 mm (SD = 0.055) (n = 15).

Fifth instar ( Figures 15-17 , 32 ): head capsule and body generally similar in color and shape to the previous instar, but shaft of the scoli reddish at the base of all five rows of spines and bluish-green between the first and second, second and third, and third and fourth rows of spines ( Figure 17 ); body with T1 and A9+10 with a bluish-green tinge. About one day before pupation, the body swollen and becomes uniformly dull green. Approximate duration: 8-22 days, including about 1 day in prepupa (average 13.1 days, n = 18). Average head capsule width: 2.62 mm (SD = 0.058) (n = 19).

**Fig 33-35.**
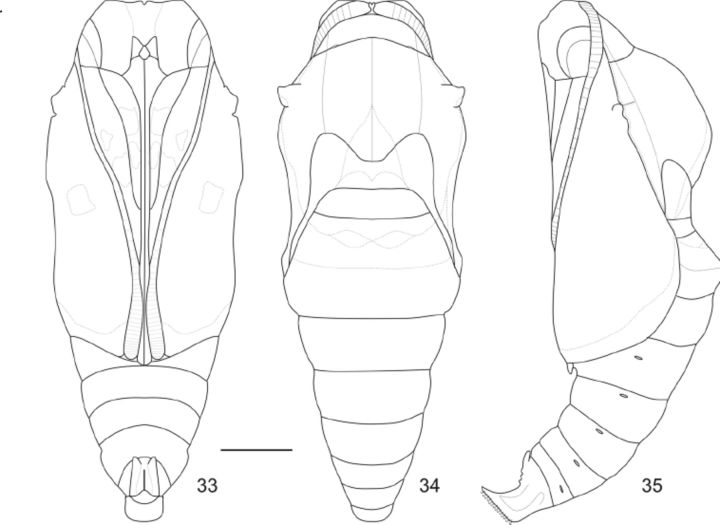
Pupae of *Callicore pygas eucale* (Fruhstorfer, 1916). 33. Ventral. 34. Dorsal. 35. Lateral. Scale bar: 0.2cm.

Pupa ( Figures 18-20 ; 33-35): mostly light green, with a dark green area on the prothorax, most of the meso and metathorax, A1, and the anterior half of A2, surrounded by brown and yellow reticulated markings; triangular light green patch on the posterior area of the mesonotum and half of the anterior medial area of the metanotum; brown and yellow reticulated markings on the head projections, basilar tubercle and along the longitudinal ridge; posterior area of A2 and A3-A9+10 light green with darker green reticulated markings; ventral area mostly green, with darker green reticulated markings and scattered brown patches on the antennae, eye cases, frons, clypeus, genae, mandibles, galeae, pro and mesothoracic legs, and the mesothoracic wing cases. Pupae flattened dorsoventrally; thorax wider at the basilar tubercle area; abdomen conical and A4-A9+10 capable of wide movements. Head projections very small; scape and pedicel dorsal, the former much larger than the latter; antennae flagellum dorsal at first, extending ventrally and posteriorly between the mesothoracic wing cases; eye cases lateral and divided in one rough and other smooth area; frons and clypeus clearly distinguishable from the genae, anterior tentorial fovea slit visible between these two areas; clypeus triangular; mandibles trapezoidal; labium pentagonal, between the mandibles and ventral to the clypeus; galeae extending between the mesothoracic legs beyond the mesothoracic wing cases and longer then the antennae. Prothorax wide and trapezoidal; mesothoracic spiracle between the prothorax and the mesothorax; mesothorax dorsally bulged at the mesonotum; basilar tubercle strongly developed, triangular and rough; longitudinal ridge ventral to the basilar tubercle, extending posteriorly to the mesothoracic wing cases; mesothoracic wing cases ventral, wing shape and venation visible; prothoracic and mesothoracic legs between the galeae and the mesothoracic wing cases, the former approximately three fourths the size of the latter; mesothoracic legs not externally visible; meta-torax ‘M’ shaped; metathoracic wing cases mostly covered, dorsally partially visible. A1– A2 totally and A3 partially ventrally covered by the thorax; A2 with a distinct ridge; first spiracle not visible; spiracles yellowish-brown and ellipsoidal; spiracles A2 and A3 dorsal, partially covered by the mesothorax, spiracles A4-A8 lateral; A5-A9+10 conical, gradually tapering posteriorly. Genital scars slits almost indiscernible on A9 (males) or A8 and A9 (females); anal scar slit distinct and surrounded by two large protuberances. Cremaster rough, large and light green; directed ventrally; ending in a flattened and strongly bilobated area with several tiny hooks. Approximate duration: 6-22 days (average 11.7 days, n = 18). Average height: 1.45cm (SD = 0.013); and width: 0.57 cm (SD = 0.035) (n = 23).

## Discussion

### General considerations

Despite the preference for *Allophylus edulis* at the study site, the use of other Sapindaceae species as host plant under laboratory conditions is not completely unexpected. *Callicore pygas eucale* reared under laboratory conditions by J.M.S. Bizarro (J.M.S. Bizarro *apud*Beccaloni et al. 2008 ) also fed on other Sapindaceae species, such as *Serjania multiflora* and an unidentified *Urvillea.* Additionally, Dias et al. (2012) reported the use of multiple species of Sapindaceae under laboratory conditions by the related species *Diaethria candrena candrena.* As suggested by Dias et al. (2012) , the preference for a host plant in a given site could be the result of the interaction with other Sapindaceae-feeding species recorded at the same location. According to Müller (1886) , eggs are laid on both sides of the leaf; however, they are uncommonly found on the upper side, but always at the leaf tip. In contrast to our observations of *C. pygas eucale,*DeVries (1987) reported that eggs of *C. atacama manova* (Fruhstorfer, 1916) are white and laid only on damaged and/or old leaves. Müller (1886) reported problems in his rearings due to high mortality of fifth instars and pupae of *C. pygas eucale.* However, of the 19 fifth instars we reared, only one failed to pupate, and of 23 pupae, only five failed to enclose.

### Morphological, taxonomic, and phylogenetic considerations

The description of the immature stages given by Müller (1886) is accurate and in accordance with our own observations. The eggs of *C. pygas eucale* are remarkably similar to those of *D. candrena candrena* ( Dias et al. 2012 ), but the latter are greenish instead of yellowish, and with horizontal ridges only close to the apex and on the vertical ridges. This is the first detailed description of the early stages for any species of *Callicore,* although Müller (1886) briefly reported early instars and behavior of *C. pygas eucale* as strikingly similar to those *of Diaethria clymena meridionalis* (Bates, 1864). In fact, the first and second instars are virtually identical to those of *D. candrena candrena* and others subspecies of *D. clymena* as well ( Barbosa et al. 2010 ; Dias et al. 2012 ), with pronounced differences appearing only from the third instar onwards, especially in the morphology of the head capsule scoli, distribution and morphology of the body scoli, and the morphologic structures and color of the pupa. For the following comparisons among fifth instars and pupae, we consulted the following sources: *C. hydaspes* (Drury, 1782), *C. cynosura cynosura* (Doubleday, 1847), *C. lyca aegina* (C. and R. Felder, 1861), *C. atacama manova, C. tolima bugaba* (Staudinger, 1876), *C. tolima peralta* (Dillon, 1948), *C. lyca aerias* (Godman and Salvin, 1883) *C. texa titania* (Salvin, 1869), *C. pitheas* (Latreille, 1813) ( Freitas 1999 ; DeVries 1987 ; Teshirogi 2007 ; Janzen and Hallwachs 2012). Data for *C. sorana sorana* (Godart, [1824]) is presented as results of personal observations of F.M.S. Dias, A.V.L. Freitas, and E.P. Barbosa (personal communication), and illustrations are provided by Freitas (1999) .

**Fig 36-44.**
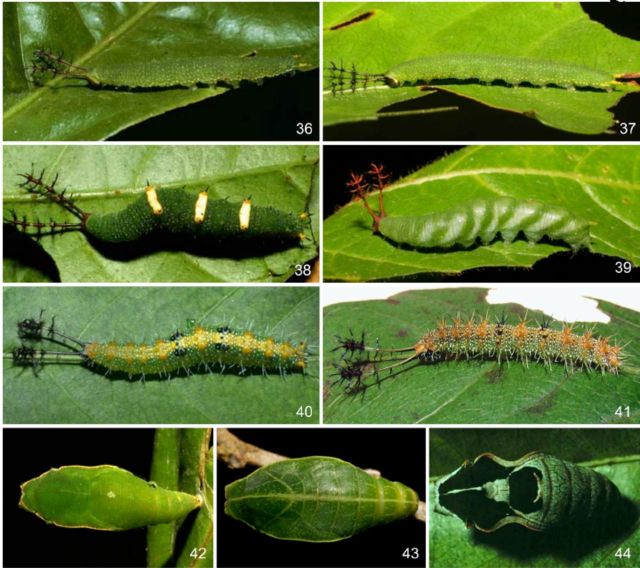
Immature stages of *Callicore* Hübner, [1819]. 36–41. Fifth instar. 36. *C. tolima bugaba* (Staudinger, 1876) (voucher code 02-SRNP-3620), lateral. 37. *C. tolima peralta* (Dillon, 1948) (voucher code 07-SRNP-41847), lateral. 38. *C. lyca aerias* (Godman & Salvin, [1883]) (voucher code 00-SRNP-11864), dorsal. 39. *C. texa titania* (Salvin, 1869) (voucher code 06-SRNP-31312), lateral. 40. *C. pitheas* (Latreille, [1813]) (voucher code 09-SRNP-55226), dorsal. 41. *C. sorana sorana* (Godart, [1824]), lateral. 42–44. Pupae, dorsal. 42. *C. tolima peralta* (Dillon, 1948) (voucher code 05-SRNP-4270). 43. *C. texa titania* (Salvin, 1869) (voucher code 08-SRNP-32149). 44. *C. pitheas* (Latreille, [1813]) (voucher code 90-SRNP-2635). Figures 36– 40 and 42–44 by Janzen and Hallwachs (2012) and Figure 41 by E. P. Barbosa.

Based on the morphology of the head capsule scoli and the abundance of body scoli of the fifth instars, species of the genus *Callicore* can be divided roughly in two major groups. The first group ( Figures 36-39 , 42-43 ), which includes *C. pygas eucale, C. atacama manova, C. tolima bugaba* ( Figure 36 ), *C. tolima peralta* (Figures 37, 42), *C. hydaspes, C. lyca aerias* ( Figure 3 8), *C. lyca aegina,* and *C. texa titania* (Figures 39, 43), is somewhat morphologically closer to *Diaethria* Billberg, 1820 and other genera of Callicorina with marked reduction of the body scoli such as *Perisama* Doubleday, 1849, *Haematera* Doubleday, 1849, and *Mesotaenia* Kirby, 1871 (see discussion in Barbosa et. al *.* 2010 and Dias et al. 2012 ). This group can be characterized by the head capsule scoli with rows of spines evenly distributed through the shaft; body scoli absent or reduced except by a pair of subdorsal scoli on the last abdominal segment, usually strongly developed and branched; with an additional dorsal scoli on A8 in some species; body usually uniformly green, often with yellowish patches; and pupa similar to species of *Diaethria* or with a two-tone green pattern.

*Callicore lyca aegina, C. lyca aerias* ( Figure 38 ), and *C. hydaspes* break the homogeneity of this group, the former two by their yellow stripes and developed pairs of subdorsal branched scoli on A2, A4, and A6; pairs of subdorsal simple scoli on A3, A5, and A7; an additional pair of branched subdorsal scoli on A8 (instead of a single A8 dorsal scolus); and the pupa with strongly developed longitudinal ridge. *Callicore hydaspes* differs by their subdorsal simple scoli on T2-T3 and dorsal and subdorsal simple scoli on A1-A8, branched on the A1 subdorsal pair ( Freitas 1999 ).

*Callicore texa titania* ( Figures 39, 43 ) differs by a number of distinctive characters: the head capsule scoli are reddish and with only four rows of spines, including the "crown"; body smooth, entirely green with whitish lateral diagonal lines; dorsal scolus on A8 and subdorsal scoli on the last abdominal segment truncated, with short thorn-like spines; and the pupa with an inconspicuous basilar tubercle, continuous with the lateral longitudinal ridge ( Figure 43 ). These characters, especially the morphology of the head capsule scoli and of the scoli on A9+10, are very similar to the observed in *Haematera pyrame pyrame* (Hübner, [1819]), another member of the Callicorina described by Müller (1886 : pl. 13 Figure 3 ).

The second group ( Figures 40-41 , 44 ) is quite homogeneous and can be characterized by the head capsule scoli with most of the spines distally displaced; distal portion of the shaft with many additional smaller spines and strongly bristled; body with large, branched and abundant dorsal, subdorsal, supraspiracular, and subspiracular scoli ( Freitas 1999 ), with variable orange or yellow patches; black patches on A4 and A6; and pupa with a two-tone green pattern. This group includes *C cynosura cy-nosura, C pitheas* ( Figure 40 ) and *C sorana sorana* ( Figure 41 ).

Based on the morphological characters of the larva presented above, *Callicore* appears to be a heterogeneous assemblage, with some species widely derived and some species morphologically very similar to each other and species of *Diaethria, Perisama, Haematera,* and *Mesotaenia* (see Müller 1886 , Attal and Crosson du Cormier 1996 , Barbosa et al. 2010 ; Greeney et al. 2010 ; Dias et al. 2012 ; Janzen and Hallwachs 2012). The presence of abundant scoli is a plesiomorphic condition within the Biblidinae ( Freitas and Brown 2004 ) found in *C cynosura cynosura, C pitheas,* and *C sorana sorana.* In a morphologic phylogeny by Freitas and Brown (2004) , *C sorana* is sister to the clade formed by *C hydaspes, D. clymena,* and *Epiphile orea* (Hübner, [1823]). Nevertheless, the position of *E. orea* is probably an artifact due to the reduced taxon sampling and scope of the study of Freitas and Brown (2004) , given that *E. orea* seems to be more closely related to species of *Temenis* Hübner, [1819] and *Nica* Hübner, [1826] (Wahlberg et al. 2009). In a molecular phylogeny by Wahlberg et al. (2009), *D. clymena* is sister to *C. tolima* (Hewitson 1851), with *Haematera* sister to this clade. Unfortunatelly, other species of *Diaethria* and *Callicore* and species of other genera of the subtribe Callicorina were not sampled. Based on the above cited assumptions, *Callicore sorana* , *C. cynosura* , and *C. pitheas* are probably sister to the rest of the species of *Callicore* . However, the comprehensiveness of *Callicore* , and the generic validity of *Diaethria, Perisama,* and *Mesotaenia* are open to discussion, since they are most likely nested within *Callicore* , and *C. texa* (Hewitson 1855) probably should be transferred to *Haematera* . Additionally, while immature of species of *Mesotaenia* resembles C *. lyca* (Doubleday, [1847]) and *C. hydaspes* ; species of *Perisama* are remarkably similar to species of *Diaethria* , *C. pygas* , *C. atacama* (Hewitson, 1851), and *C. tolima* (Hewitson, 1851).

It is expected that further information on immature stages of other species could shed a light on the taxonomy of the Callicorina, which is based chiefly on the color and pattern of the wings ( Attal and Crosson du Cormier 1996 ). We remind that only superficial characters could be compared in the present study, and detailed descriptions as presented by Barbosa et al. (2010) and Dias et al *.* (2012) certainly will improve comparative studies in the future. Additionally, descriptions of morphology and host plant use for species of some genera of Callicorina without any information of the immature stages will be helpful to extract further characters for taxonomy and phylogeny ( Freitas and Brown 2004 ).

## References

[R1] Attal S Crosson du Cormier A . 1996 . The genus Perisama. Sciences Nat.

[R2] Beccaloni GW Hall SK Viloria AL Robinson GS . 2008 . Host-plants of the Neotropical Butterflies: A Catalogue / Catálogo de las Plantas Huésped de las Mariposas Neotropicales, S.E.A./RIBES-CYTED/The Natural History Museum/ Instituto Venezolano de Investigaciones Científicas.

[R3] Barbosa EP Kaminski LA Freitas AVL . 2010 . Immature stages of the butterfly *Diaethria clymena janeira* (Lepidoptera: Nymphalidae: Biblidinae). *Zoologia* 27 : 696-702.

[R4] D’Abrera B . 1987 . Butterflies of the Neotropical region, part IV, Nymphalidae (Partim). Hill House.

[R5] D’Almeida RF . 1922 . Mélanges Lépidoptérologiques. Études sur les Lépidoptères Du Brésil. R. Friedländer & Sohn.

[R6] Descimon H . 1986 . L évolution de la coloration chez les Charaxidae néotropicaux: stratégies adaptatives et cladogenèse (Lepidoptera Rhopalocera). *Bulletin de la Société zoologique de France* 111 : 261-295.

[R7] DeVries P . 1987 . The butterflies of Costa Rica and their natural history, Papilionidae, Pieridae, Nymphalidae. Princeton University Press.

[R8] Dias FMS Carneiro E Casagrande MM Mielke OHH . 2012 . Biology and external morphology of immature stages of the butterfly, *Diaethria candrena candrena.* Journal of Insect Science 12(9). Available online: www.insectscience.org/12.9 10.1673/031.012.0901 PMC3465927 22943597

[R9] Freitas AVL . 1999 . Nymphalidae (Lepidoptera): filogenia incluindo caracteres de imaturos, com testes de planta hospedeira. PhD Thesis, Instituto de Biologia, Universidade Estadual de Campinas.

[R10] Freitas AVL Brown Jr. KS . 2004 . Phylogeny of the Nymphalidae (Lepidoptera). *Systematic Biology* 53 : 363-383. 10.1080/10635150490445670 15503668

[R11] Greeney HF Dyer LA DeVries PJ Walla TR Salazar LV Simbaña W Salgaje L . 2010 . Early stages and natural history of *Perisama oppelii* (Latreille, 1811) (Nymphalidae, Lepidoptera) in eastern Ecuador. *Kempffiana* 6 : 16-30.

[R12] Hinton HE . 1946 . On the morphology and nomenclature of setae of the lepidopterous larvae, with notes on the phylogeny of the Lepidoptera. *Transactions of the Royal Entomological Society London* 97 : 1-35.

[R13] Huertas-Dionisio M . 2006 . Estados inmaturos de Lepidoptera (XXVI). Quetotaxia de las patas anales de las orugas (Insecta: Lepidoptera). *SHILAP Revista de Lepidopterología* 34 : 213-228.

[R14] Janzen DH Hallwachs W. 2010 . Dynamic database for an inventory of the macrocaterpillar fauna, and its food plants and parasitoids, of Área de Conservación Guanacaste (ACG), northwestern Costa Rica. Available online: http://janzen.sas.upenn.edu

[R15] Lamas G . 2004 . Biblidinae. In: Lamas G, Heppner JB, Editors. *Checklist: Part 4A. Hesperioidea-Papilionoidea. Atlas of Neotropical Lepidoptera, Volume 5A.* pp. 234-247. Scientific Publishers.

[R16] Mosher E . 1916 . A classification of the Lepidoptera based on characters of the pupa. *Bulletin of the Illinois State Laboratory of Natural History* 12 : 1-165.

[R17] Müller W . 1886 . Südamerikanische Nymphalidenraupen. Versuch eines natürlichen Systems der Nymphaliden. *Zoologische Jahrbücher* 1 : 417-678.

[R18] Neild A . 1996 . The butterflies of Venezuela, Part 1: Nymphalidae I (Limenitidinae, Apaturinae, Charaxinae). A comprehensive guide to the identification of adult Nymphalidae, Papilionidae, and Pieridae. Meridian Publishing.

[R19] Peterson A . 1962 . Larvae of insects. An introduction to Neartic species. Part I. Lepidoptera and plant infesting Hymenoptera. Edwards Brothers.

[R20] Scoble M . 1992 . The Lepidoptera, form, function and diversity. Natural History Museum Publications/Oxford University Press.

[R21] Stehr FW . 1987 . Order Lepidoptera. *In:* Stehr, F.W, Editor. *Immature insects.* pp. 288–305. Kendall/Hunt.

[R22] Teshirogi M . 2007 . The splendid Biblidinae in the Neotropical region. *Butterflies (Teinopalpus)* 47 : 30 – 44 .

[R23] Vegilante F Hasenfuss I. 2012 . Morphology and diversity of exocrine glands in lepidopteran larvae. *Annual Review of Entomology* 57 : 187 – 204 . 10.1146/annurev-ento-120710-100646 21910636

[R24] Wahlberg N Leneveu J Kodandaramaiah U Peña C Nylin S Freitas AVL Brower AVZ 2009 . Nymphalid butterflies diversify following near demise at the cretaceous/tertiary boundary. *Proceedings of the Royal Society (B)* 276 : 4295-4302. 10.1098/rspb.2009.1303 PMC2817107 19793750

